# Patient Sex and Origin Influence Distribution of Driver Genes and Clinical Presentation of Paraganglioma

**DOI:** 10.1210/jendso/bvae038

**Published:** 2024-02-29

**Authors:** Susan Richter, Nicole Bechmann

**Affiliations:** Institute for Clinical Chemistry and Laboratory Medicine, Faculty of Medicine and University Hospital Carl Gustav Carus, Technische Universität Dresden, 01307 Dresden, Germany; Institute for Clinical Chemistry and Laboratory Medicine, Faculty of Medicine and University Hospital Carl Gustav Carus, Technische Universität Dresden, 01307 Dresden, Germany

**Keywords:** pheochromocytoma, germline, somatic pathogenic variants, Asian, European

## Abstract

**Context:**

Sexual and ancestral differences in driver gene prevalence have been described in many cancers but have not yet been investigated in pheochromocytoma and paraganglioma (PPGL).

**Objective:**

This study aims to assess whether sex and ancestry influence prevalence of PPGL driver genes and clinical presentation.

**Methods:**

We conducted a retrospective analysis of patients with PPGL considering studies from 2010 onwards that included minimal data of type of disease, sex, mutated gene, and country of origin. Additional features were recorded when available (age, tumor location, bilateral or multifocal, somatic or germline, and metastatic disease).

**Results:**

We included 2162 patients: 877 in Europe and 757 in Asia. Males presented more often with germline pathogenic variants (PVs) in genes activating hypoxia pathways (*P* = .0006) and had more often sympathetic paragangliomas (*P* = .0005) and metastasis (*P* = .0039). On the other hand, females with PPGLs due to *MAX* PVs were diagnosed later than males (*P* = .0378) and more often developed metastasis (*P* = .0497). European but not Asian females presented more often with PPGLs due to PVs in genes related to kinase signaling (*P* = .0052), particularly *RET* and *TMEM127*. Contrary to experiences from Europe, Asian patients with PPGL due to PVs in kinase signaling genes *NF1*, *HRAS*, and *FGFR1* showed a high proportion of sympathetic tumors, while European patients almost exclusively had adrenal tumors (*P* < .005).

**Conclusion:**

Personalized management of patients with PPGL might benefit from considering sexual and ancestral differences. Further studies with better clinically aligned cohorts from various origins are required to better dissect ancestral influences on PPGL development.

Sex disparities are documented for cancer incidence and mortality across many entities, reflecting in part different occupational risk exposures and lifestyle choices [1]. Additionally, molecular processes are regulated differently in males and females, eg, through sex-biased epigenetic marks [2] or interaction of specific single nucleotide polymorphisms with sex hormone signaling [3] that lead to differential gene expression, including differential efficiency in DNA repair and immune response [4]. Sex bias was described in whole genome data from pan-cancer studies highlighting differences in mutation frequency of driver genes, density of single nucleotide variants, and copy number variation of specific chromosomes [5, 6]. The observed alterations were distinct between cancer types. Recently, we demonstrated that both sexes have different susceptibilities to the occurrence of adrenal tumors [7].

Pheochromocytoma and paragangliomas (PPGL) are a group of neoplasms that originate from adrenal medullary [pheochromocytoma (PCC)], sympathetic [paraganglioma (PGL)], or parasympathetic [head and neck paraganglioma (HNP)] chromaffin cells. Genetic drivers of PPGLs can be explained in about 80% of cases, half of which are caused by germline pathogenic variants (PVs) in 1 of more than 20 susceptibility genes [8]. The most commonly mutated genes, germline and somatic, include succinate dehydrogenase subunit genes (*SDHA*, *SDHB*, *SDHC, SDHD*), Von Hippel-Lindau (*VHL*) tumor suppressor, neurofibromatosis 1 (*NF1*), *RET* proto-oncogene, transmembrane protein 127 (*TMEM127*), MYC associated factor X (*MAX*), endothelial PAS domain protein 1 (*EPAS1,* encoding hypoxia-inducible factor (HIF) 2α), *HRAS* proto-oncogene, and fibroblast growth factor receptor 1 (*FGFR1*). Other genes occur more rarely, including but not limited to fumarate hydratase (*FH*), SDH assembly factor (*SDHAF2*), and isocitrate dehydrogenase 1 (*IDH1*). Based on their transcriptional profile, PPGLs are divided into distinct clusters. Cluster 1 PPGLs are characterized by HIF signaling, which is either caused by mutations in genes involved in mitochondrial metabolism and subsequent action of oncometabolites [cluster 1A, including *SDHx* (*SDHA/B/C/D/AF2*), *FH*, *IDH1*] or genes directly influencing HIF2α expression and stabilization (cluster 1B, including *VHL*, *EPAS1*), whereas cluster 2 includes PPGLs with aberrations in the kinase signaling pathway (*RET*, *NF1*, *TMEM127*, *MAX*, *HRAS*, *FGFR1*) [9]. A close correlation exists between the genotype and the phenotype of PPGLs; eg, in that cluster 1 PPGLs have a significantly higher risk of metastasis than cluster 2 PPGLs [10, 11].

Previously, we identified differences in the prevalence and clinical presentation of PVs in specific genes between a European and a Chinese PPGL cohort [12]. Sexual dimorphism of genetic drivers, on the other hand, has not yet been investigated systematically in a large data set of patients with PPGL. To examine sexual and ancestral differences on a bigger scale, we conducted a retrospective data compilation of all recent genetic studies of PPGL. This analysis will give more insights into specific features of patients with genetic subtypes of PPGL and aims to support efforts toward personalized PPGL management.

## Materials and Methods

**Figure 1.**
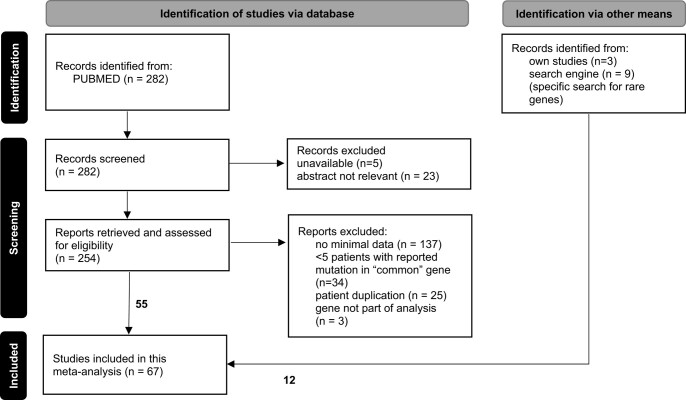
Procedure of study inclusion.

A retrospective data analysis was conducted by searching in PUBMED with the following string: ((paraganglioma[Title]) OR (pheochromocytoma[Title]) OR (phaeochromocytoma[Title]) OR (paragangliomas[Title]) OR (pheochromocytomas[Title]) OR (phaeochromocytomas[Title])) AND ((mutation[Title]) OR (mutations[Title]) OR (gene[Title]) OR (genes[Title]) OR (genetics[Title]) OR (genomics[Title]) OR (genetic[Title]) OR (genomic[Title])) AND (English[Language]) NOT (review[Publication Type]) NOT (case report) NOT (a patient[Title]) NOT (cells[Title]) NOT (cell[Title]) NOT (expression[Title]) NOT (single-nuclei[Title]) NOT (single-cell[Title]) NOT (case[Title]). Reviews, case reports, articles in languages other than English, and articles published before 2010 were excluded. The search term (performed April 4, 2023) yielded 282 articles that were collected manually by the first author and evaluated for suitability by both authors (Fig. 1) [13]. Papers were included when minimal data (type of disease, sex, mutated gene, country of origin) from at least 5 patients were available. Patients with gene variants of unknown significance were excluded. If no classification was given in the publication, variants were checked in the ClinVar and LOVD databases. Variants classified as pathogenic or likely pathogenic were summarized as PVs for the purpose of this publication. For rare genes, also papers with fewer than 5 patients were included. A number of studies were checked for potential overlaps to exclude duplication of patients. This procedure yielded 55 papers. Additionally, 12 studies were included either due to availability of our published data or based on specific keyword searches for rare genes, including *FH*, *SDHA*, *SDHAF2*, *IDH1*, and *EPAS1* [13]. Finally, 67 publications with a total of 2162 patients, who presented with PPGL, were included in this analysis [14]. Besides minimal data, the following features were collected: age at diagnosis, tumor location, bilateral or multifocal disease, somatic or germline status, and presence of metastases. To prevent patient duplication, patients from the same center with the same sex, age, and gene mutation were only recorded once. Origin or ancestry was stated according to the country of origin of the study, if not otherwise specified in the publication. Patients from China, South Korea, Japan, and India were summarized as Asian. Patients from Belgium, Czech Republic, France, Germany, Hungary, Ireland, Italy, The Netherlands, Norway, Poland, Portugal, Russia, Spain, Sweden, Turkey, and the United Kingdom were called European. Patients from Australia, Brazil, Canada, Columbia, Israel, New Zealand, Saudi Arabia, and the United States were not categorized in either group due to their more diverse and mixed origins.

Statistical analyses of compiled data were performed with JMP Pro17. Categorical data were compared by Pearson's chi-squared test, whereas age as a continuous variable was assessed by the Kruskal–Wallis test. Differences were considered significant when *P* < .05. Patients with partially missing data were excluded from analyses, with patient numbers recorded in parentheses.

## Results

### Distributions of Genes With PVs Between Sexes and Patient Origin

Our data set contained 2162 patients [14] who presented with PPGL and in whom a germline or somatic PV in a PPGL susceptibility gene from the cluster 1 or 2 branch was detected [11, 12, 15-79]. The overall percentage of females was 51.1% (1104/2162). European patients were significantly more often female (54.8%, *P* = .0075) than Asian patients (48.2%). Age at diagnosis, on the other hand, did not differ between Asian and European patients (Table 1).

**Table 1.** Sexual differences in tumor location, gene clusters, and metastasis between European and Asian patients (displayed as % females)

		Asia	Europe	*P*-value
	Age at diagnosis*^a^*	40 (4-82, 757)	41 (6-83, 852)	.3322
Cluster	1A	44.2 (87/197)	51.8 (236/456)	.0749
	1B	54.8 (98/179)	47.8 (55/115)	.2463
	2	47.2 (180/381)	62.1 (190/306)	.0001
Location	PCC	48.7 (190/390)	56.4 (237/420)	.0281
	PGL	44.6 (94/211)	42.0 (66/157)	.6307
	HNP	51.9 (14/27)	58.2 (153/263)	.5267
	PPGL+	38.9 (7/18)	67.7 (23/34)	.0458
	Metastasis	41.2 (28/68)*^b^*	47.7 (51/107)*^b^*	.4006

Abbreviations: HNP, head and neck paraganglioma; PCC, pheochromocytoma; PGL, abdominal/thoracic paraganglioma; PPGL+, combination of at least 2 different tumor locations.

*P*-values calculated according to Pearson's chi-squared test.

^*a*^Median (range, n).

^*b*^Metastatic disease was reported to a similar extent, with 10.9% (68/624) for Asians and 13.3% (107/804) for Europeans.

**Figure 2.**
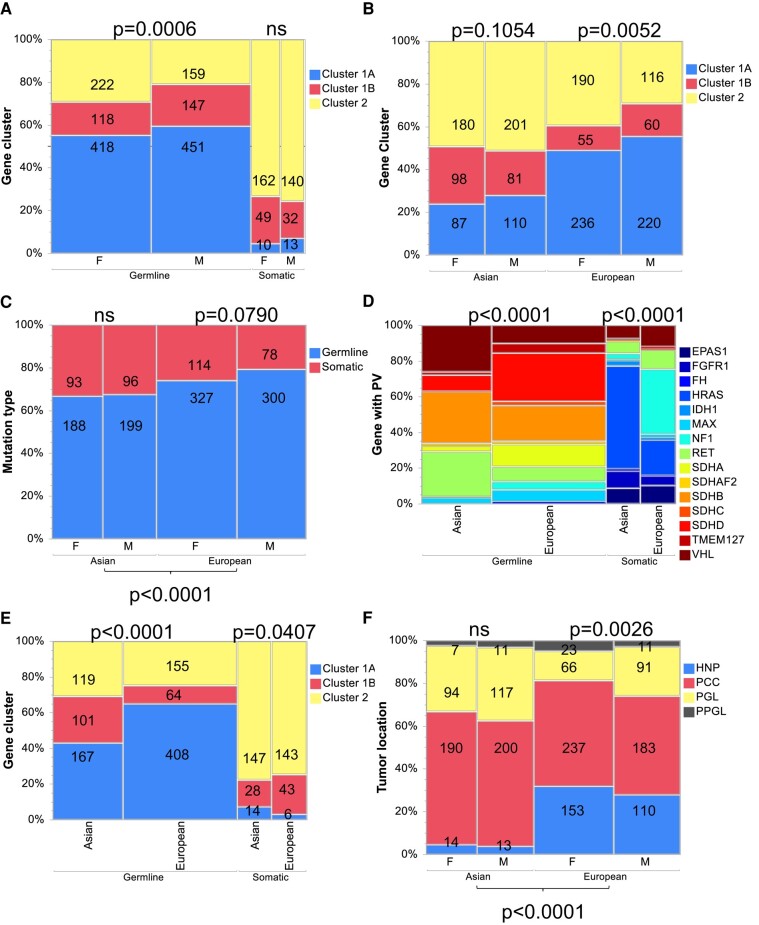
Clinical features of patients with PPGL according to sex and patient origin. Abbreviations: F, female; HNP, head and neck paraganglioma; M, male; ns, not significant; PCC, pheochromocytoma; PGL, paraganglioma of thorax or abdomen; PPGL, at least 2 of the stated tumor locations occurred in the same patient; PV, pathogenic variant.

Germline PVs in cluster 1A and 1B genes were overall more abundant in males than females compared to germline PVs in cluster 2 genes that occurred more often in females than males (*P* = .0006, Fig. 2A). Especially, germline PVs in *RET* and *TMEM127* were associated with female sex and PVs in *SDHB* and *VHL* with male sex (Table 2). Sexual distribution of all reported somatic PVs did not differ between clusters (Fig. 2A). However, female sex was significantly more common in patients with somatic *EPAS1* PV, whereas male sex was more often reported in patients with *FGFR1* PV (Table 2). Somatic *NF1* PVs showed a trend toward higher representation in females.

**Table 2.** Percentage of female patients with pathogenic variants in PPGL susceptibility genes overall and according to patient origin

Gene	Sex				Origin (as % female)		
	% female	*P*-value	*P*-value germline	*P*-value somatic	Asia	Europe	*P*-value
*EPAS1*	81.0 (64/79)	<.0001	.2568 (5/7)	<.0001 (36/44)	90.5 (38/42)	69.6 (16/23)	.0316
FGFR1*	37.8 (28/74)	.0364	­		34.4 (22/64)	60.0 (6/10)	.1202
*FH*	60.9 (14/23)	.2971	.1573 (12/18)		33.3 (1/3)	60.0 (6/10)	.4164
HRAS*	53.3 (80/150)	.4142			50.5 (55/109)	61.5 (24/39)	.2339
IDH1*	33.3 (3/9)	.3173	­	­	28.6 (2/7)	50.0 (1/2)	.5708
*MAX*	49.2 (31/63)	.8997	.7855 (26/54)	.6547 (2/5)	43.8 (7/16)	53.3 (24/45)	.5102
*NF1*	55.7 (97/174)	.1295	.4751 (27/49)	.0809 (50/84)	48.9 (23/47)	60.2 (62/103)	.1968
*RET*	56.3 (152/270)	.0385	.0178 (120/206)	.7389 (19/36)	51.1 (69/135)	65.3 (49/75)	.0465
*SDHA*	49.0 (50/102)	.8430	.9191 (48/97)	­	43.8 (7/16)	51.3 (41/80)	.5839
*SDHAF2* ^▴^	55.0 (11/20)	.6547		­	83.3 (5/6)	38.5 (5/13)	.0685
*SDHB*	44.0 (201/457)	.0101	.0047 (188/435)	­	41.0 (50/122)	47.4 (63/133)	.3052
*SDHC*	57.5 (23/40)	.3428	.6015 (18/33)	­	50.0 (1/2)	61.9 (13/21)	.7417
*SDHD*	53.5 (159/297)	.2230	.3266 (141/266)	­	47.4 (18/38)	54.3 (107/197)	.4320
*TMEM127*	67.6 (50/74)	.0025	.0030 (48/71)	­	40.0 (4/10)	72.7 (24/33)	.0571
*VHL*	43.4 (139/328)	.0058	.0463 (113/258)	.2059 (16/40)	45.0 (63/140)	42.4 (39/92)	.6953

Abbreviations: PPGL, pheochromocytoma and paraganglioma.

All *P*-values were calculated according to Pearson’s chi-squared test; patient numbers are stated in parentheses. *P*-values are only given for groups of at least 5 patients. Only somatic (*) or germline (*^▴^*) variants known in PPGL.

Female predominance occurred in European patients among cluster 2 genes (*P* = .0052), while males reported more often with cluster 1A PVs (Fig. 2B, Table 1). The latter was also observed as a trend in Asian patients with females presenting relatively more often with cluster 1B but not cluster 2 PVs (*P* = .1064). Especially, *RET* and *TMEM127* PVs occurred more frequently among European females compared to Asian females (Table 2). *EPAS1* PVs showed female dominance in Europe and Asia but were stronger in Asian patients, where 90% were females compared to 70% in European patients. The frequency of all reported germline versus somatic PVs did not differ between sexes neither in the European nor the Asian population, but the overall proportion of germline PVs was higher in the European cohort (*P* < .0001, Fig. 2C).

Prevalence of reported driver genes differed according to patient origin (Fig. 2D). Germline PVs in cluster 1A genes were more abundant among European than Asian patients, whereas cluster 1B and cluster 2 genes showed higher prevalence in Asian than European patients (*P* < .0001, Fig. 2E). Among Asian patients, most prevalent genes with germline PV were *SDHB* (28.9%), *VHL* (26.1%), and *RET* (25.1%) compared to *SDHD* (27.2%), *SDHB* (19.7%), *SDHA* (12.5%), and *VHL* (10.2%) in European patients. In contrast, PVs in somatic cluster 1B genes had a somewhat higher frequency in European than Asian patients (Fig. 2E). Somatic *HRAS* (57.6%), *FGFR1* (9.5%), *EPAS1* (9.0%), and *VHL* (7.4%) were most common in Asian patients, whereas *NF1* (36.5%), *HRAS* (19.8%), *VHL* (12.0%), *RET* (10.9%), and *EPAS1* (10.4%) were most frequently affected by somatic PVs in European patients.

### Differential Clinical Presentation in Males and Females

Clinical features differed between sexes in that males presented at a younger age and had more often abdominal or thoracic PGL and metastases, while females were more often diagnosed with HNP (Table 3). Additionally, gene-specific differences in clinical presentation were evaluated. PVs in *FGFR1* caused predominantly PCC in females but more often PGL in males (Table 4). *SDHA* PVs were almost 60% associated with HNP in females but mostly with PGL in males. Although *SDHB* PVs caused predominantly PGL in both sexes, females showed a higher proportion of HNPs. For patients with PVs in *SDHD*, HNP followed by PGL were the dominant presentations in both sexes, but males had more often PCC compared to females (7.3% vs 1.3%, *P* = .0277). Multifocality differed between sexes only among patients with *SDHB* PVs (females: 11.8% vs males: 23.3%, *P* = .0254). Metastatic disease was reported more often in males with *SDHA* PVs than females (28.6% vs 12.2%, *P* = .0450) but more often in females with *MAX* PVs than males (19.4% vs 3.3%, *P* = .0497, Table 5). Interestingly, females with *MAX* PV presented at an older age than males (Table 6). Age between females with and without reported metastasis did not differ (*P* = .1397). On the other hand, females with *RET* PV were younger at diagnosis than males, and these differences could not be explained by differences in the number of germline or somatic mutations; rather, female patients with *RET* germline PV were diagnosed earlier than corresponding males (*P* = .0012).

**Table 3.** Comparison of clinical features between sexes

Feature	Female	Male	*P*-value
Age at diagnosis (years)*^a^*	40 (6-83, 1092)	39 (4-81, 1044)	.0435
Germline PVs (%)	77.4 (758/979)	80.4 (757/942)	.1152
PCC (%)	55.9 (582/1042)	53.3 (536/1006)	.2422
PGL (%)	28.4 (296/1042)	35.6 (358/1006)	.0005
HNP (%)	20.0 (208/1042)	15.4 (155/1006)	.0070
Bilateral tumors (%)	24.4 (164/663)	23.7 (145/611)	.6760
Multifocal tumors (%)	17.6 (102/580)	21.2 (110/518)	.1262
Metastasis (%)	15.3 (150/981)	20.3 (197/971)	.0039

Abbreviations: HNP, head and neck paraganglioma; PCC, pheochromocytoma; PGL, paraganglioma of thorax or abdomen; PV, pathogenic variant.

*P*-values were calculated according to the Kruskal–Wallis test for continuous variables and Pearson’s chi-squared test for categorical variables. Percentages are displayed with patient numbers in parentheses.

^*a*^Median (range, n).

**Table 4.** Differences in tumor location between sexes according to gene

Gene	Females				Males				*P*-value
	PCC	PGL	HNP	PPGL+	PCC	PGL	HNP	PPGL+	
*EPAS1*	51.6 (33/64)	40.6 (26/64)	­	7.8 (5/64)	46.6 (7/15)	40.0 (6/15)	­	13.3 (2/15)	.7879
*FGFR1*	78.6 (22/28)	22.4 (6/28)	­	­	30.4 (14/46)	69.6 (32/46)	­	­	<.0001
*FH*	61.5 (8/13)	23.1 (3/13)	0 (0/13)	15.4 (2/13)	44.4 (4/9)	22.2 (2/9)	22.2 (2/9)	11.1 (1/9)	.3551
*HRAS*	76.3 (61/80)	23.8 (18/80)	­	­	68.6 (48/70)	31.4 (22/70)	­	­	.2925
*IDH1*	­	66.7 (2/3)	33.3 (1/3)	­	­	100 (6/6)	­	­	.1336
*MAX*	87.1 (27/31)	0 (0/31)	­	12.9 (4/31)	96.7 (29/30)	3.3 (1/30)	­	­	.0798
*NF1*	97.0 (95/97)	2.1 (2/97)	­	­	96.0 (72/7)	4.0 (3/75)	­	­	.4531
*RET*	98.4 (124/126)	1.6 (2/126)	­	­	98.9 (93/94)	1.1 (1/94)	­	­	.7405
*SDHA*	16.3 (8/48)	22.5 (11/49)	59.2 (29/49)	2.0 (1/49)	24.5 (12/49)	42.7 (21/49)	30.6 (15/49)	2.0 (1/49)	.0388
*SDHAF2*	­	­	100 (7/7)	­		­	100 (8/8)	­	—
*SDHB*	9.5 (18/190)	64.2 (122/190)	24.7 (47/190)	1.6 (3/190)	9.7 (24/247)	72.1 (178/247)	13.8 (34/247)	4.5 (11/247)	.0134
*SDHC*	4.6 (1/22)	36.4 (8/22)	59.1 (13/22)	­	11.8 (2/17)	17.7 (3/17)	70.6 (12/17)		.3609
*SDHD*	1.3 (2/157)	30.6 (48/157)	61.8 (97/157)	6.4 (10/157)	7.3 (10/138)	27.5 (38/138)	54.4 (75/138)	10.9 (15/138)	.0277
*TMEM127*	91.8 (45/49)	­	4.1 (2/49)	4.1 (2/49)	95.7 (22/23)	4.4 (1/23)	­	­	.2580
*VHL*	79.0 (98/124)	9.7 (12/124)	0.8 (1/124)	­	88.8 (159/179)	4.5 (8/179)	0.6 (1/179)	6.2 (11/179)	.1301

Abbreviations: HNP, head and neck paraganglioma; PCC, pheochromocytoma; PGL, abdominal/thoracic paraganglioma; PPGL+, combination of at least 2 different tumor locations.

*P*-values calculated according to Pearson’s chi-squared test.

**Table 5.** Reported metastatic disease (in %) according to sex and origin

Gene	Sex			Origin		
	Female	Male	*P*-value	Asia	Europe	*P*-value
*EPAS1*	5.4 (3/56)	7.1 (1/14)	.7968	—	—	—
*FGFR1*	0 (0/28)	2.2 (1/46)	.4322	1.6 (1/64)	0 (0/10)	.6906
*FH*	28.6 (4/14)	33.3 (3/9)	.8086	33.3 (1/3)	40.0 (4/10)	.8351
*HRAS*	5.1 (4/79)	2.9 (2/70)	.4942	3.7 (4/109)	5.3 (2/38)	.6690
*IDH1*	—	—	—	—	—	—
*MAX*	19.4 (6/31)	3.3 (1/30)	.0497	14.3 (2/14)	11.1 (5/45)	.7484
*NF1*	4.1 (4/97)	5.3 (4/75)	.7087	0 (0/46)	3.9 (4/102)	.1733
*RET*	4.8 (6/125)	2.2 (2/91)	.3173	3.7 (3/81)	2.7 (2/75)	.7133
*SDHA*	12.2 (6/49)	28.6 (14/49)	.0450	25.0 (3/12)	18.8 (15/80)	.6108
*SDHAF2*	—	—	—	—	—	—
*SDHB*	49.7 (86/173)	57.7 (138/239)	.1063	45.4 (44/97)	43.9 (50/114)	.8269
*SDHC*	16.7 (3/18)	18.8 (3/16)	.8736	0 (0/1)	22.2 (3/16)	.6333
*SDHD*	11.6 (15/129)	12.6 (15/119)	.8136	10.0 (3/30)	7.6 (12/157)	.6632
*TMEM127*	4.1 (2/49)	0 (0/23)	.3258	0 (0/8)	6.1 (2/33)	.4753
*VHL*	9.1 (11/121)	7.4 (13/176)	.5964	6.4 (7/109)	8.7 (8/92)	.5411

*P*-values according to the Kruskal–Wallis test. Percentages are displayed with numbers in parentheses.

**Table 6.** Age (in years) differences between sexes and according to origin

Gene	Sex			Origin		
	Female	Male	*P*-value	Asia	Europe	*P*-value
*EPAS1*	46 (12-78, 62)	51 (10-73, 15)	.6571	47 (32-69, 42)	46 (13-78, 21)	.3392
*FGFR1*	49 (33-80, 28)	47 (32-72, 46)	.2647	47 (32-72, 64)	54 (41-80, 10)	.1614
*FH*	45 (20-69, 14)	41 (6-77, 9)	.7050	36 (30-48, 3)	39 (6-70, 10)	.8656
*HRAS*	53 (25-79, 78)	55 (31-76, 69)	.8812	53 (25-75, 109)	55 (31-79, 36)	.3051
*IDH1*	58 (54-61, 3)	70 (49-79, 6)	.1213	62 (49-79, 7)	69 (61-78, 2)	.5582
*MAX*	36 (16-58, 31)	29 (13-57, 32)	.0378	31 (16-53, 16)	32 (13-58, 45)	.8375
*NF1*	52 (15-83, 96)	48 (16-80, 75)	.0903	48 (26-82, 47)	53 (27-83, 100)	.1154
*RET*	34 (15-77, 147)	41 (14-76, 117)	.0026	39 (14-71, 135)	43 (18-77, 69)	.0388
*SDHA*	40 (11-81, 50)	43 (14-68, 51)	.7753	41 (13-64, 16)	42 (11-81, 79)	.8814
*SDHAF2*	34 (23-52, 11)	36 (21-47, 9)	.6480	36 (32-49, 6)	36 (21-52, 13)	.8605
*SDHB*	34 (7-81, 200)	31 (6-80, 250)	.1149	27 (7-74, 122)	36 (9-81, 127)	.0078
*SDHC*	46 (16-71, 23)	49 (32-81, 17)	.1507	36 (19-54, 2)	51 (16-71, 21)	.4131
*SDHD*	35 (10-68, 159)	37 (14-71, 138)	.1513	33 (18-61, 38)	39 (11-71, 197)	.1415
*TMEM127*	39 (20-72, 50)	44 (21-76, 24)	.3287	38 (33-52, 10)	44 (20-76, 33)	.4371
*VHL*	26 (5-79, 138)	25 (4-69, 186)	.2535	25 (4-69, 140)	26 (7-70, 88)	.9121

*P*-values according to the Kruskal–Wallis test. Medians are displayed with ranges and numbers in parentheses. This analysis does not differentiate between germline and somatic variants.

### Differential Clinical Presentation in Asians and Europeans

Tumor localization differed significantly between Asian and European patients (Fig. 2F, Table 7). For the latter, HNP was reported proportionally more often than in the Asian population, whereas Asian patients had more often PGL and PCC compared to European patients. Bilateral tumors were recorded more often in European than Asian patients. Metastases were reported to a similar extent overall and in PCC (Table 7). PGLs were reported more often in association with metastasis in European than Asian patients, but for individual genes no differences were apparent (Table 5).

**Table 7.** Comparison of clinical features between Asian and European patients

Feature	Asia	Europe	*P*-value
Age at diagnosis*^a^*	40 (4-82, 757)	41 (6-83, 852)	.3322
PCC (%)	63.2 (408/646)	51.7 (452/874)	<.0001
PGL (%)	35.1 (227/646)	21.2 (185/874)	<.0001
HNP (%)	4.8 (31/646)	31.2 (273/874)	<.0001
Bilateral tumors (%)	21.2 (141/666)	27.1 (125/461)	.0209
Multifocal tumors (%)	15.8 (54/342)	19.8 (109/551)	.1332
Metastasis (%)	10.9 (68/624)	13.3 (107/804)	.1682
Metastasis by location*^b^*:			
PCC (%)	5.8 (22/379)	6.7 (28/416)	.5912
PGL (%)	20.0 (41/205)	35.5 (55/155)	.0010

Abbreviations: HNP, head and neck paraganglioma; PCC, pheochromocytoma; PGL, abdominal/thoracic paraganglioma.

*P*-values calculated according to the Kruskal–Wallis test for continuous variables and Pearson’s chi-squared test for categorical variables. Percentages are displayed with patient numbers in parentheses.

^*a*^Median (range, n).

^*b*^HNP was excluded due to imbalanced numbers between races.

As stated earlier, a larger proportion of germline variants, especially those in cluster 1A genes, was recorded in European compared to Asian patients (Fig. 2C). Assessing these differences for each individual gene in association with tumor location shows that proportionally more HNPs were reported for European patients with PVs in *SDHA*, *SDHB*, *SDHC*, and *SDHD* than for Asian patients (Table 8). On the other hand, *NF1*, *HRAS*, and *FGFR1* PVs were more often associated with PGL in the Asian population, whereas European patients predominantly presented with PCC. A number of *EPAS1*-related patients (17.4%) had PPGLs at different locations, but no such patients have been reported in Asia so far.

**Table 8.** Percentage of patients with pathogenic variants in PPGL susceptibility genes according to origin and tumor location

Gene	Asia				Europe				*P*-value
	PCC	PGL	HNP	PPGL+	PCC	PGL	HNP	PPGL+	
*EPAS1*	54.8 (23/42)	45.2 (19/42)	­	­	52.2 (12/23)	30.4 (7/23)	­	17.4 (4/23)	.0171
*FGFR1*	42.2 (27/64)	57.8 (37/64)	­	­	90.0 (9/10)	10.0 (1/10)	­	­	.0049
*FH*	66.7 (2/3)	33.3 (1/3)	­	­	55.6 (5/9)	11.1 (1/9)	11.1 (1/9)	22.2 (2/9)	.6338
*HRAS*	64.2 (70/109)	35.8 (39/109)	­	­	94.9 (37/39)	5.1 (2/39)	­	­	.0002
*IDH1*	­	­	100 (7/7)	­	­	50.0 (1/2)	50.0 (1/2)	­	.0472
*MAX*	92.9 (13/14)	7.1 (1/14)	­	­	91.1 (41/45)	­	­	8.9 (4/45)	.1074
*NF1*	91.3 (42/46)	8.7 (4/46)	­	­	100 (102/102)	­	­	­	.0025
*RET*	96.5 (82/85)	3.5 (3/85)	­	­	100 (75/75)	­	­	­	.1005
*SDHA*	33.3 (4/12)	58.3 (7/12)	8.3 (1/12)	­	18.8 (15/80)	26.3 (21/80)	52.5 (42/80)	2.5 (2/80)	.0260
*SDHAF2*	­	­	100 (1/1)	­		­	100 (13/13)	­	—
*SDHB*	17.5 (18/103)	74.8 (77/103)	4.9 (5/103)	2.9 (3/103)	6.8 (9/132)	47.7 (63/132)	41.7 (55/132)	3.8 (5/132)	<.0001
*SDHC*	100 (1/1)	­	­	­	9.5 (2/21)	23.8 (5/21)	66.7 (14/21)		.0362
*SDHD*	8.3 (3/36)	19.4 (7/36)	55.6 (20/36)	16.7 (6/36)	3.6 (7/197)	24.4 (48/197)	67.5 (133/197)	4.6 (9/197)	.0232
*TMEM127*	100 (8/8)	­	­	­	84.9 (28/33)	3.0 (1/33)	6.1 (2/33)	6.1 (2/33)	.7101
*VHL*	84.4 (97/115)	7.8 (9/115)	­	7.8 (9/115)	83.7 (77/92)	7.6 (7/92)	2.2 (2/92)	6.5 (6/92)	.4530

Abbreviations: HNP, head and neck paraganglioma; PCC, pheochromocytoma; PGL, abdominal/thoracic paraganglioma; PPGL+, combination of at least 2 different tumor locations.

*P*-values calculated according to Pearson’s chi-squared test.

Asian patients were diagnosed earlier than European patients with *SDHB*-related tumors, which was not due to differences in the germline status (Table 6). Asian patients presented significantly younger with *RET*-associated tumors than European patients; however, there were significantly more individuals with germline variants in the Asian cohort.

## Discussion

PPGL are one of the best genetically characterized neoplasms and contain the highest proportion of germline PVs among all tumor entities. Here, we show the extent of sexual and ancestral differences across well-known PPGL susceptibility genes and that the same underlying driver gene can result in distinct clinical presentations between sexes and ancestral groups, eg, in respect to tumor location and age of diagnosis. Insights into specific factors that influence clinical presentation may improve individualized care of PPGL patients, thus leading to better patient outcomes. We consider our analysis to be a starting point for further clinical and mechanistic investigations, and we aim to raise awareness about sexual and ancestral differences.

Similar to our study on sex differences in adrenal diseases [7], we found a higher percentage of females with PPGL in European compared to Asian patients. Additionally, the European cohort contained a much higher fraction of HNPs and germline cluster 1A genes than the Asian one. We previously reported that patients with HNP but without *SDHx* PV were more frequently female [78]. In the present cohort of patients with defined genetic drivers, HNPs due to cluster 1A gene PVs occurred also more often in females, explaining in part the higher percentage of females in Europe. Nevertheless, female sex in Europe was significantly associated with cluster 2 and not cluster 1A gene PVs. The reason for the higher number of female patients with PPGL in Europe is unknown, but it appears to be a more general phenomenon that occurs across PCC [7] and HNP [78] but not PGL as our analysis showed. Behavioral or environmental factors might play a role but have not been studied in PPGL. Males in general had a higher proportion of PGL, cluster 1A gene PVs, were slightly younger at diagnosis, presented more often with multifocality in association with *SDHB* PVs, and had a higher rate of metastasis. These findings are consistent with a study analyzing disease-specific survival in patients with PPGL that showed male sex, younger age, extra-adrenal location, multifocality, and *SDHB* PVs to be associated with metastasis [80]. Sex-dependent distortion of *SDHB* penetrance toward males was noted in previous research [81, 82]. Accelerated disease progression was associated with male sex in a previous study [83].

Our analysis also shows sexual differences among patients with *SDHA* PVs in that males had more often PGL and metastatic disease than females. A trend of male predominance among patients with *SDHA* PVs was noted in a previous report [75]. Knowledge about sexual disparities could be implemented into screening guidelines to ensure that tumors with high metastatic potential are caught early in their development. Experts are currently in disagreement about the usefulness of PPGL screening in *SDHA* PV carriers due to their low penetrance [77]. Our findings suggest that sex differences should be considered in this discussion and that males might benefit from earlier or more frequent screening.

Female predominance among carriers of the cluster 2 gene *TMEM127* PV was reported previously [84] and confirmed in this analysis. In addition, European females with PPGL were more often carriers of *RET* germline PVs, and they presented earlier with PPGL than males. While sexual differences in chromatin organization and DNA repair might be plausible explanations for somatic PVs and somatic second hits in tumor suppressor genes, another mechanism must be at play for dominant germline PVs in, eg, *RET*. In this case, developmental advantages or disadvantages of RET activation in combination with hormone action might occur. Estrogen responsive transcriptional enhancers in *RET* were described in connection with breast cancer [85] and may play a role in the observed sex differences for PPGL.

The only cluster 2 gene with male predominance was *FGFR1*. Amplification and overexpression are associated with resistance to estrogen receptor-targeted therapy in luminal breast cancer [86, 87] and indicate a regulatory connection of *FGFR1* and estrogen signaling. Furthermore, our analysis showed that females with PPGL due to *MAX* PV were diagnosed later than males and presented more often with metastatic disease. This result was unrelated to mutation type, as most *MAX* PVs occurred in the germline. Additionally, later tumor diagnosis in women did not appear to be causal for developing metastatic disease, since there was no age difference between females with and without metastases. Whether differences in severity or type of symptoms could account for the differences in the age of diagnosis is unknown. Publications have stated general differences in the reporting and presentation of clinical symptoms between sexes [88-90], but nothing is known in regard to specific PPGL driver genes or differences between Asians and Europeans. Differences in clinical presentation as a result of sex and origin could be due to differences in cellular origin or differences in the microenvironment, which might favor the development of tumors from certain cell populations.

Two-thirds of European patients with somatic PVs in *EPAS1* were female, while in Asians this number was even higher. It is known that hypoxic exposure, either through high altitude [91, 92] or disease, such as congenital heart disease [72, 93] or sickle cell disease [94], increases the risk for somatic *EPAS1* PVs and subsequent PPGL development. Prevalence of congenital heart disease does not differ between sexes [95], indicating that other factors contribute to increased prevalence in females. Sex differences were reported in hemoglobin levels, vasodilation, oxygen delivery and uptake, and their response to hypoxic episodes [96-99]. Such distinctions may be responsible for a sex-dimorphic susceptibility to acquire somatic *EPAS1* PVs. Germline *EPAS1* PVs also show a trend toward female predominance [100]; however, due to low numbers, significance was not reached with our analysis. Estrogen receptor α was shown to function as a transcriptional repressor of *EPAS1* in breast cancer cells [101] and might be involved in sexual dimorphism of susceptibility toward *EPAS1* PVs.

Tumorigenesis due to *VHL* loss follows a similar mechanism to *EPAS1* PVs, since VHL ubiquitinates HIFα for degradation. Nevertheless, more men than women were reported to have PPGL due to *VHL* PVs irrespective of germline or somatic status. The same observation was made in a meta-analysis focused specifically on PPGL patients with VHL disease [102]. Other studies found that the involved organs in germline *VHL*-mutated patients are not associated with sex but rather with age and mutation type [103-105]. On the other hand, males suffer from a higher tumor burden of central nervous system hemangioblastomas, while females show faster growth rates of *VHL*-related clear cell renal cell carcinomas [106, 107]. As penetrance appears to be unaffected by sex, other factors, such as susceptibility to de novo mutation or embryonic selection, may play a role.

There are only few publications addressing ancestral differences in patients with PPGL [12]. Our analysis recorded differences in the prevalence of somatic drivers in that European patients more often presented with *VHL* and *NF1* PVs than Asian patients, whereas *HRAS* and *FGFR1* were dominant in the Asian population and other than for European patients associated with PGL. We confirm and extend results from Jiang et al, who first reported on Sino-European differences. Interestingly, another study identified that PVs in another growth factor receptor *EGFR* are more common in lung cancer among Asian than White or Black patients [108]. On the other hand, *KRAS* mutations occurred to a lesser extent in Asian patients. Our analysis found differences in the age of diagnosis for PPGLs caused by *SDHB* PVs, with Asians presenting earlier than Europeans with a tumor. For *SDHB* PV carriers, this difference may be caused by the higher percentage of HNPs among Europeans compared to Asians, as HNPs generally do not produce catecholamines and therefore present with fewer signs and symptoms. The lack of HNPs in Asian patients might be an artifact due to differences in reporting strategies and screening priorities between countries and should be viewed with caution.

The presented analysis of published patients with PPGL and known driver gene aimed at reducing bias as much as possible, since studies were only omitted if minimal data was not available or when considerable risk of patient duplication was eminent, eg, in case of 2 large-scale studies from the same clinical center. Nevertheless, bias is present through varied availability of screening and genetic testing procedures; clinical protocols, eg, for workups on multifocality; and data reporting between Asian and European countries. This may be a reason for the underrepresentation of Asian patients with HNP in our cohort and indicates that more studies investigating HNPs in the Asian population are needed. Overall, more studies from European [38] than from Asian [19] countries were available and included in this retrospective analysis, resulting in higher patient numbers for Europeans (877 vs 757). This study reported on the country of origin of patients but does not reflect ethnic differences that occur in these countries or between people from different countries of Europe or Asia. Further limitations have to be considered when interpreting results about metastatic disease, since follow-up was not part of the inclusion criteria for this meta-analysis. Hence, the rate of metastatic disease in this population is most likely underestimated [80, 109]. The year 2010 was chosen as a cut-off for publications, since the majority of common PPGL susceptibility genes (*RET*, *NF1*, *VHL*, *SDHx*, TMEM127) was known by then and a few others (*MAX*, *EPAS1*, *FH*) were discovered within the next few years. Nevertheless, strategies for genetic testing changed over the years. Targeted Sanger sequencing and multiplex-ligation dependent probe amplification were used in 2010, while next-generation sequencing with the capability of multiple gene analyses was the technique of choice by 2017 [8]. This process may have been somewhat delayed in Asia compared to Europe, as reports from Europe often originate from specialized referral centers with other diagnostic capabilities than hospitals from lower-income countries. Additionally, the present analysis might suffer from influences of specific gene variants or founder mutations in some populations that might impact generalization about PVs in a particular gene.

This retrospective analysis establishes for the first time that sex and genetic background influence clinical features of PPGLs in a PV-dependent manner. Our observations suggest that factors beyond genotype influence the development of PPGL with sex and genetic origin being involved in this process. A better understanding of the underlying mechanisms and their clinical consequences may lead to measures that delay or perhaps even prevent tumor development in PV carriers and may help to explain differences in disease penetrance. One such measure could be that males with *SDHx* PVs are screened more frequently and earlier than females; especially for *SDHA* PVs, earlier disease onset was found in males. Furthermore, in Asian patients with elevated plasma metanephrine levels, not only adrenal tumors but also sympathetic PGL should be considered, as adrenergic PGL occurs often compared to European patients. Our findings could lead to improved personalized management strategies and should be viewed as a starting point for future investigations. Some of the presented results could be used as a basis for mechanistic studies that with the help of model organisms can investigate whether molecular processes of PPGL development differ between sexes or origins for particular driver genes. Sex-specific alterations in molecular pathways may modulate the response to targeted therapies and could be implemented into personalized treatment schemes. Cohort studies dissecting inheritance patterns in multiple generations or clinical as well as behavioral features that might influence susceptibility to somatic mutations should provide further information in the future.

## Data Availability

Raw data were deposited online at Zenodo: https://zenodo.org/records/10695390; https://zenodo.org/records/10695469.
